# Supplementary material for: Whole-Genome Sequencing-Based Screening of MRSA in Patients and Healthcare Workers in Public Hospitals in Benin
Source: Microorganisms. 2023 Jul 31;11(8):1954. doi: 10.3390/microorganisms11081954 (PMC10459514; doi:10.3390/microorganisms11081954)
Supplement: Supplementary file 1 [file microorganisms-11-01954-s001.zip › microorganisms-2500979-supplementary.pdf]

Supplementary Material

Tables

Table S1: Mammalian reference genomes included in the Kraken 2 database

**Species**	**RefSeq accession**
Ades aegypti	GCF_002204515
Aedes albopictus	GCF_006496715
Anas platyrhynchos	GCF_015476345
and Stomoxys calcitrans	GCF_001015335
Apis mellifera	GCF_003254395
Bos taurus	GCF_002263795
Capra hircus	GCF_001704415
Cavia porcellus	GCF_000151735
Chlorocebus sabaeus	GCF_015252025
Columba livia	GCF_000337935
Culex quinquefasciatus	GCF_015732765
Equus caballus	GCF_002863925
Gallus gallus	GCF_000002315
Homo sapiens	GCF_000001405
Ixodes scapularis	GCF_002892825
Meleagris gallopavo	GCF_000146605
Mesocricetus auratus	GCF_000349665
Mus musculus	GCF_000001635
Numida meleagris	GCF_002078875
Ovis aries	GCF_002742125
Rattus norvegicus	GCF_015227675
Sus scrofa	GCF_000003025

This table lists the reference genomes included in the in-house Kraken 2 database. The first column lists the species. The second column lists the RefSeq accession number.

Table S2: Overview of the generated WGS datasets

Isolate	Run	QC status	SRA accession
32	B	Low depth	SRR23547053
32	C	OK	SRR23547083
562	B	Low depth	SRR23547099
562	C	OK	SRR23547078
680	B	Contaminated	SRR23547095
680	C	OK	SRR23547075
905	B	OK	SRR23547093
905	C	OK	SRR23547073
907	B	Low depth	SRR23547091
907	C	OK	SRR23547072
961	A	Contaminated	SRR23547104
961	B	Low depth	SRR23547090
961	C	OK	SRR23547071
994	F	OK	SRR23547058
995	F	OK	SRR23547057
996	F	OK	SRR23547056
998	E	OK	SRR23547066
1002	F	OK	SRR23547065
1043	D	OK	SRR23547069
1052	F	OK	SRR23547064
1053	F	OK	SRR23547063
1057	F	OK	SRR23547062
1398	E	Contaminated	SRR23547067
1401	F	OK	SRR23547061
1405	F	OK	SRR23547060
1684	B	Low depth	SRR23547103
1684	C	OK	SRR23547089
1701	B	Contaminated	SRR23547092
1701	C	Fragmented	SRR23547088
2007	B	Contaminated	SRR23547081
2007	C	OK	SRR23547087
2090	B	Contaminated	SRR23547070
2090	C	OK	SRR23547086
2656	B	Low depth	SRR23547059
2656	C	OK	SRR23547085
2777	B	Low depth	SRR23547055
2777	C	OK	SRR23547084
2855	B	Contaminated	SRR23547054
3247	B	Fragmented	SRR23547052
3247	C	OK	SRR23547082
3326	B	OK	SRR23547102
3326	C	Contaminated	SRR23547080
4101	B	OK	SRR23547101
4101	C	OK	SRR23547079
5232	B	Low depth	SRR23547100
5232	D	OK	SRR23547068
5729	B	OK	SRR23547098

5729	C	OK	SRR23547077
6020	B	Contaminated	SRR23547097
6512	B	Low depth	SRR23547096
6512	C	Low depth	SRR23547076
7111	B	Low depth	SRR23547094
7111	C	OK	SRR23547074

The first and second columns list the isolate and run names, respectively. The third column indicates if the dataset passed the quality checks described in the Material & Methods. Note that the quality checks are evaluated sequentially (e.g., a dataset that is labeled as 'Contaminated' could also have low depth and/or a fragmented assembly). The last column lists the SRA accession number.

Table S3: Overview of the sequenced datasets and the selected high-quality dataset per isolate

isolate	Run A	Run B	Run C	Run D	Run E	Run F
32	-	low depth	OK	-	-	-
562	-	low depth	OK	-	-	-
680	-	contaminated	contaminated	-	-	-
905	-	OK (not used)	OK	-	-	-
907	-	low depth	OK	-	-	-
961	contaminated	low depth	OK	-	-	-
994	-	-	-	-	-	OK
995	-	-	-	-	-	OK
996	-	-	-	-	-	OK
998	-	-	-	-	OK	-
1002	-	-	-	-	-	OK
1043	-	-	-	OK	-	-
1052	-	-	-	-	-	OK
1053	-	-	-	-	-	OK
1057	-	-	-	-	-	OK
1398	-	-	-	-	contaminated	-
1401	-	-	-	-	-	OK
1405	-	-	-	-	-	OK
1684	-	low depth	OK	-	-	-
1701	-	contaminated	fragmented	-	-	-
2007	-	contaminated	OK	-	-	-
2090	-	contaminated	OK	-	-	-
2656	-	low depth	OK	-	-	-
2777	-	low depth	OK	-	-	-
2855	-	contaminated	-	-	-	-
3247	-	fragmented	OK	-	-	-
3326	-	OK	contaminated	-	-	-
4101	-	OK (not used)	OK	-	-	-
5232	-	low depth	-	OK	-	-
5729	-	OK (not used)	OK	-	-	-
6020	-	contaminated	-	-	-	-
6512	-	low depth	low depth	-	-	-
7111	-	low depth	OK	-	-	-

This table provides an overview of all WGS datasets that were generated. Datasets that passed the QC checks are marked as ‘OK’. When multiple datasets from one isolate passed all QC checks, the least fragmented (i.e., highest N50) was retained for the analysis. Datasets that passed the QC checks but that were not used afterwards are marked as ‘not used’.

Table S4: Read-trimming statistics

Isolate	Read pairs in	Read pairs after trimming	Forward only surviving	Reverse only surviving	Dropped pairs
2019_071_3326	389,318	376,363	9,451	1,496	2,008
2020_040_1684	407,374	386,680	16,891	1,743	2,060
2020_040_2007	313,007	288,293	21,724	982	2,008
2020_040_2090	457,715	423,759	30,201	1,321	2,434
2020_040_2656	338,855	318,532	17,322	1,149	1,852
2020_040_2777	321,816	306,014	12,891	1,469	1,442
2020_040_32	434,252	418,745	11,515	2,159	1,833
2020_040_3247	542,805	517,681	20,798	1,984	2,342
2020_040_4101	416,700	393,212	20,009	1,477	2,002
2020_040_562	407,244	382,023	21,410	1,541	2,270
2020_040_5729	524,811	493,420	26,420	2,036	2,935
2020_040_7111	318,791	296,238	18,725	2,026	1,802
2020_040_905	252,794	239,684	10,679	944	1,487
2020_040_907	399,918	381,236	14,981	2,037	1,664
2020_040_961	249,510	232,972	13,799	1,128	1,611
2020_041_1043	623,472	603,544	14,316	3,357	2,255
2020_041_5232	505,589	484,350	17,919	1,448	1,872
2020_045_998	377,646	356,546	18,541	938	1,621
2022_017_1002	841,302	779,525	51,569	4,104	6,104
2022_017_1052	798,722	739,538	49,355	3,698	6,131
2022_017_1053	671,853	621,664	41,517	3,542	5,130
2022_017_1057	659,337	621,433	29,130	4,694	4,080
2022_017_1401	658,773	613,191	36,785	3,970	4,827
2022_017_1405	769,974	710,812	50,019	3,550	5,593
2022_017_994	797,064	743,059	44,471	4,187	5,347
2022_017_995	849,255	792,694	45,058	5,552	5,951
2022_017_996	829,225	768,673	49,538	4,393	6,621

The first column lists the isolate name. The second and third columns list the number of read pairs before and after trimming, respectively. The fourth and fifth columns list the number of forward and reverse orphaned reads after trimming, respectively. The sixth column lists the number of read pairs that were dropped.

Table S5: *De novo* assembly statistics

Isolate	Nb. of contigs	Total length	N50	Median depth
2019_071_3326	61	2,854,071	73,523	51
2020_040_1684	33	2,869,415	273,468	57
2020_040_2007	24	2,746,284	257,536	46
2020_040_2090	36	2,810,804	239,711	62
2020_040_2656	27	2,725,313	335,890	52
2020_040_2777	39	2,866,367	147,242	42
2020_040_32	48	2,865,914	140,837	53
2020_040_3247	30	2,788,144	257,715	73
2020_040_4101	35	2,850,650	153,642	58
2020_040_562	33	2,855,097	227,416	53
2020_040_5729	28	2,770,352	288,972	71
2020_040_7111	32	2,849,716	209,691	40
2020_040_905	38	2,754,061	153,059	35
2020_040_907	34	2,756,916	176,059	52
2020_040_961	32	2,901,686	173,257	32
2020_041_1043	41	2,804,121	128,990	82
2020_041_5232	39	2,884,896	336,748	67
2020_045_998	32	2,846,679	273,462	55
2022_017_1002	35	2,826,466	288,051	111
2022_017_1052	30	2,845,033	275,437	115
2022_017_1053	42	2,857,223	273,393	98
2022_017_1057	28	2,859,387	364,492	94
2022_017_1401	29	2,877,784	300,121	87
2022_017_1405	26	2,835,369	275,295	113
2022_017_994	23	2,877,542	326,564	113
2022_017_995	28	2,893,609	326,670	113
2022_017_996	34	2,887,721	235,828	115

The first column lists the isolate name. The second, third, and fourth column list the number of contigs, N50, and total assembly length, respectively. These metrics were calculated by QUAST. The fifth column lists the median read depth, determined by mapping the processed reads against the assembly using Bowtie2.

Table S6: Sequence types, *spa* types, and SCC*mec* types

isolate	ST	*spa* type	SCC*mec* type
2020_040_7111	8	t121	IVa(2B)
2020_040_961	8	t121	IVa(2B)
2020_040_1684	8	t1476	IV(2B&5)(1)
2020_040_2090	8	t1476	IV(2B&5)(1)
2020_040_32	8	t1476	IV(2B&5)(1)
2020_041_5232	8	t1476	IV(2B&5)(1)
2020_045_998	8	t1476	IV(2B&5)(1)
2022_017_1052	8	t1476	IV(2B&5)(1)
2022_017_1053	8	t1476	IV(2B&5)(1)
2022_017_1057	8	t1476	IV(2B&5)(1)
2022_017_1401	8	t1476	IV(2B&5)(1)
2022_017_1405	8	t1476	IV(2B&5)(1)
2022_017_994	8	t1476	IV(2B&5)(1)
2022_017_995	8	t1476	IV(2B&5)(1)
2022_017_996	8	t1476	IV(2B&5)(1)
2019_071_3326	121	t314	IVa(2B)
2020_040_2777	121	t314	IVa(2B)
2020_040_4101	121	t314	IVa(2B)
2020_040_562	121	t314	IVa(2B)
2020_041_1043	121	t314	IVa(2B)
2020_040_905	152	t1096	Va(5C2)
2020_040_907	152	t1096	Va(5C2)
2020_040_2656	152	t355	XIII(9A)(2)
2020_040_2007	152	t4690	Vc(5C2&5)
2020_040_3247	152	t4690	Vc(5C2&5)
2020_040_5729	772	t657	V(5C2)
2022_017_1002	789	t091	V(5C2&5)

(1) The ccrA2, dmecR1 and CCB2 genes were missing in these isolates, and prediction was, therefore, solely based on homology to the complete SCCmec cassette which was covered for ~55%. (2) No match found based on homology for whole cassette, prediction was solely on genes.

This table lists the results of the WGS-based typing. The first column lists the isolate name. The second, third, and fourth columns list the sequence types, *spa* types, and SCC*mec* types, respectively.
